# Case report: detection of the identical virus in a patient presenting with severe fever with thrombocytopenia syndrome encephalopathy and the tick that bit her

**DOI:** 10.1186/s12879-018-3092-y

**Published:** 2018-04-17

**Authors:** Uh. Jin Kim, Dong-Min Kim, Seong Eun Kim, Seung Ji Kang, Hee-Chang Jang, Kyung-Hwa Park, Sook In Jung

**Affiliations:** 1 Chonnam National University Medical School, Gwang-ju, Republic of Korea; 2 Chosun University College of Medicine, Gwangju, Republic of Korea

**Keywords:** SFTS, SFTSV, Encephalopathy, Tick, Transmission, Vector

## Abstract

**Background:**

Severe fever with thrombocytopenia syndrome (SFTS) is an emerging tick-borne disease. *Haemophysalis longicornis* ticks have been considered the vector of severe fever with thrombocytopenia syndrome virus (SFTSV). However, clear data on the transmission of SFTS from ticks to humans are limited.

**Case presentation:**

We report an 84-year-old woman who presented with fever and altered mentality, which was confirmed as SFTS with encephalopathy by reverse-transcription polymerase chain reaction in blood and cerebrospinal fluid. The SFTSV was also identified in the tick that bit her, *H. longicornis*. Phylogenetic analyses indicated that the SFTSV from the patient and the tick was identical. The patient gradually recovered with treatments of corticosteroids and immunoglobulin.

**Conclusion:**

These findings provide further evidence of SFTS viral transmission from *H. longicornis* to human.

## Background

Severe fever with thrombocytopenia syndrome (SFTS) was first recognized in 2009, and the responsible virus (SFTSV) was first isolated from a patient’s blood in China in 2010 [1, 2]. *Haemaphysalis longicornis* ticks collected from domestic animals in the areas where affected patients lived were found to contain SFTSV RNA [1, 2]. The RNA sequences of these viruses were very closely related, but not identical, to the SFTSV isolated in samples obtained from the patients, which suggests viral transmission from ticks to humans [1, 2]. Herein, we describe a case of SFTS encephalopathy and detection of the identical SFTSV from human blood and cerebrospinal fluid (CSF) and from a tick that bit the patient.

## Case presentation

**Fig. 1:**
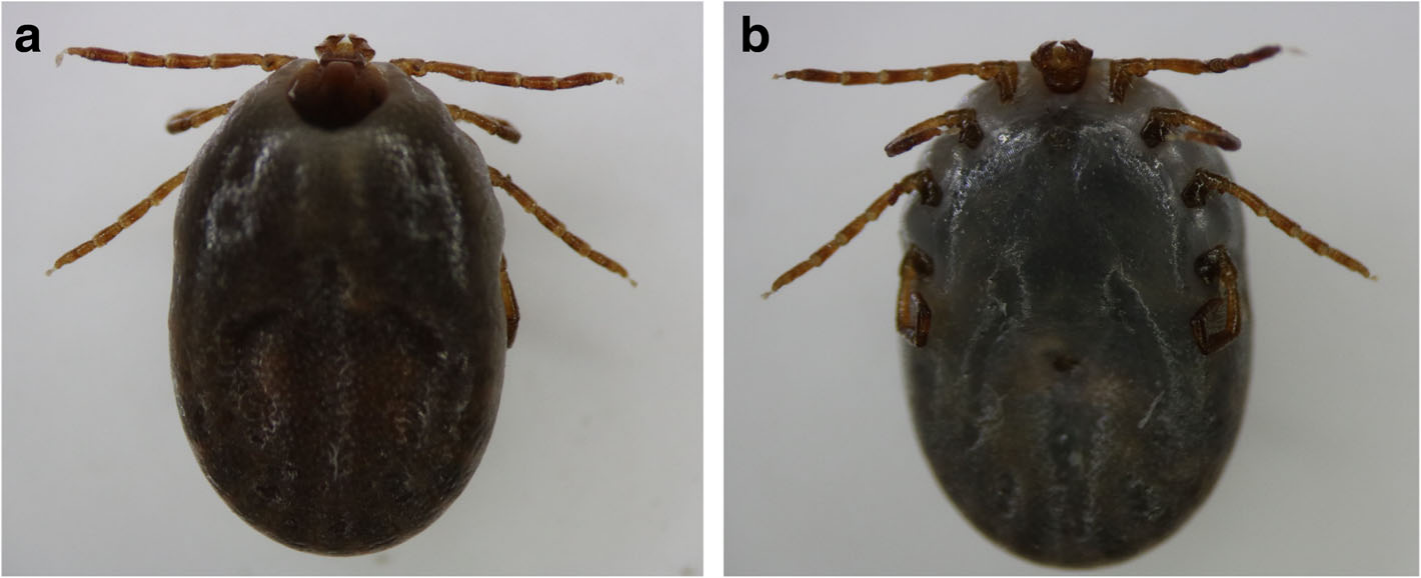
Dorsal (**a**) and ventral (**b**) images of the *Haemaphysalis longicornis* specimen retrieved from the patient

On June 29, 2017, an 84-year-old woman was presented at Chonnam National University Hospital located in southwestern part of Republic of Korea, with fever, diarrhea, and general weakness for 6 days; she was admitted to the hospital. She had a history of treated scrub typhus infection more than 5 years ago. Meanwhile she had a small yard in front of her house, where she grew vegetables. On admission, the patient showed stuporous mentality, with a Glasgow coma scale (GCS) score of 8. She had a blood pressure of 110/70 mmHg, pulse rate of 80 beats/min, respiratory rate of 24 breaths/min, and body temperature of 38.1 °C. Oxygen saturation on room air was 98%. A biting tick was noted on the patient’s right popliteal fossa (Fig. 1). No skin rash or eschar was observed on physical examination. Laboratory examinations revealed a white blood cell count of 1200/mm^3^ (normal range 4800~ 10,800/ mm^3^), hemoglobin level of 12.4 g/dL (normal range 12~ 18 g/dL), and platelet count of 23,000/mm^3^ (normal range 130,000~ 450,000/mm^3^). The serum levels of C-reactive protein, ferritin, aspartate aminotransferase (AST), alanine transaminase (ALT), and lactate dehydrogenase (LDH) were 0.6 mg/dL (normal < 0.6 mg/dL), 14,578.6 ng/mL (normal range 4.63~ 274.66 ng/mL), 166 U/L (normal range 10~ 37 U/L), 45 U/L (normal range 10~ 37 U/L), and 1546 U/L (normal range 218~ 472 U/L), respectively. The serum coagulation profiles of activated partial thromboplastin time, prothrombin time, and fibrinogen were 51.0 s (normal range 26.5~ 41 s), 11.0 s (normal range 9.8~ 13 s), and 149.6 mg/dL (normal range 180~ 350 mg/dL), respectively. An initial brain computed tomography examination revealed no signs of cerebral hemorrhage or acute large-vessel infarction. CSF examination showed a leukocyte count of 2/mm^3^ (normal ≤ 5/mm^3^), protein concentration of 34 mg/dL (normal range 20~ 45 mg/dL), and glucose concentration of 66 mg/dL (normal range 40~ 80 mg/dL).

Evaluations to determine the microbiological cause of fever included blood cultures, reverse-transcription polymerase chain reaction (RT-PCR) to detect the SFTSV in blood, indirect immunofluorescence to detect scrub typhus and Hantaan virus, and passive hemagglutination testing for *Leptospira*. We conducted RT-PCR to amplify M-segment using forward Primer MF3 (5’-GATGAGATGGTCCATGCTGATTCTAA-3′) and reverse primer MR2 (5’-CTCATGGGGTGGAATGTCCTCAC-3′) [3], while RT-nested PCR to amplify S-segment of SFTS virus using two set of primers NP-2F (5′-CATCATTGTCTTTGCCCTGA-3′) and NP-2R (5′- AGAAGACAGAGTTCACAGCA-3′) for the first round of PCR (amplicon size: 461 bp) and N2F (5′-AAY AAG ATC GTC AAG GCA TCA-3′) and N2R (5′-TAG TCT TGG TGA AGG CAT CTT-3′) for the nested PCR (amplicon size: 346 bp) [4]. Moreover, real-time RT-PCR targeting at the S segment was performed using forward primer SFTS-SQ-F (5’-ACCTCTTTGACCCTGAGTTWGACA-3′), reverse primer SFTS-SQ-R (5’-CTGAAGGAGACAGGTGGAGATGA-3′), and TaqMan MGB (minor groove binder) probe SFTS-SQ-P (5’-FAM-TGCCTTGACGATCTTA-NFQ-MGB -3′) coupled with FAM (Fluorescein amidite) [5]. The results of RT-PCR for the SFTSV in blood were positive. A standard calibration curve was generated for target quantification using 10-fold serial dilutions of plasmid in which the M segment of the SFTSV was cloned. Quantitative RT-PCR for the SFTSV in plasma yielded a Ct value of 24.71, with 2 × 10^5^/mL viral RNA copies. Results of blood culture and tests for scrub typhus, Hantaan virus, and *Leptospira* were negative. Based on the clinical manifestations, detection of a biting tick, and laboratory findings, SFTS-associated encephalopathy was diagnosed. On day 2, the patient received intravenous immunoglobulin (1 g/kg/24 h in three doses) and dexamethasone (10 mg/m^2^) for three days [6].

The intact tick retrieved from the patient was washed three times with 70% alcohol and then three times with distilled water prior to making tick lysate. The lysate was prepared using phosphate-buffered saline (PBS) with 10% fetal bovine serum and 1 mL antibiotic penicillin-streptomycin solution/100 mL PBS (final concentration, 100 IU/mL penicillin and 100 μg/mL streptomycin). RNA was extracted using a Viral Gene Spin™ Viral RNA extraction kit (iNTRON Biotechnology, Korea) according to the manufacturer’s instructions. SFTSV from the tick was detected by RT-PCR. Quantitative RT-PCR for SFTSV in the tick sample was positive, with a Ct value of 32.82 and 2 × 10^3^/mL viral RNA copies.

On day 5 of admission, although defervescence was achieved and the AST, ALT, LDH, and ferritin levels had begun to decrease, the GCS score remained in the range of 8–10. On day 6 of admission, follow-up CSF examination revealed a leukocyte count of 1/mm^3^, protein concentration of 45 mg/dL, and glucose concentration of 73 mg/dL. Although the viral load in the plasma decreased (Ct value, 36.83; viral RNA copies, 10^2^/mL), the SFTSV was detected in CSF by real-time RT-PCR (Ct value, 33.61; viral RNA copies, 10^3^/mL). In order to avoid the cross contamination, RNA extraction from Blood, CSF and tick samples and the PCR were performed in subsequently different days and reconfirmed.

**Fig. 2:**
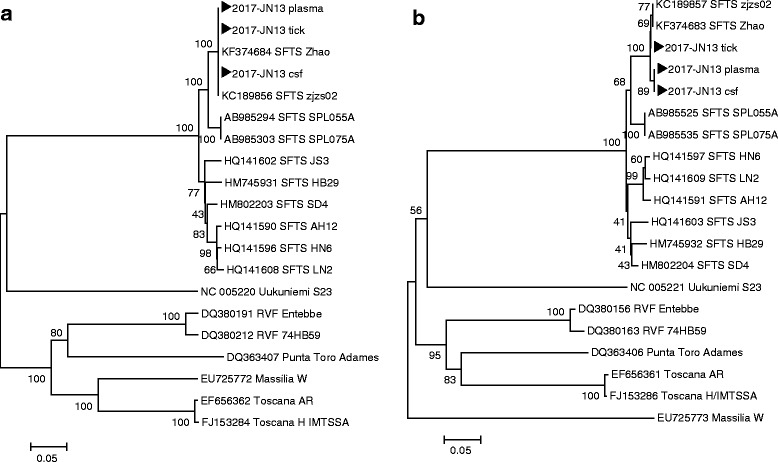
SFTSV phylogenetic tree based on the partial medium (M) segments (477 bp) (**a**) and the partial small (S) segment (324 bp) (**b**) of isolates showing positivity for SFTSV (black arrow) from the patient’s blood and cerebrospinal fluid and from the tick, compared to SFTSV sequences from GenBank. The GenBank accession number is indicated. The scale bar indicates a sequence distance of 0.1 nucleotide substitutions per site

To compare the nucleotide sequence of SFTSV samples from human and tick, phylogenetic analyses based on the partial medium (M) (477 bp) and small (S) segments (324 bp) were performed [4]. The sequences in the partial M segments of SFTSV from the patient’s plasma and CSF and that from the tick were 100% identical to the isolates from China (accession numbers: KC189856 SFTS zjzs02 and KF374684 SFTS Zhao (Fig. 2a). In addition, the sequences in the partial S segments of SFTSV from patient’s samples and that from tick shared 99.4% identity to each other (Fig. 2b). For sequence alignment LaserGene Program (DNASTAR, Madison, WI) was used and phylogenetic tree was constructed by the neighbor joining (N-J) method using ClustalX software program. Representative SFTSV sequences from GenBank, including SFTS isolates from other countries, and previously identified SFTS sequences from Republic of Korea were included in the phylogenetic analysis. Sequences obtained in this study were submitted to NCBI Genbank, and the accession numbers assigned are as following: MH114954-MH114959. These findings indicated that the SFTSV from the patient’s plasma and CSF and that from the tick were identical.

The patient’s recovery was slow; on day 10 of admission, her GCS score was 12, with a near-alert mental state. After recovering from SFTS, she remained in the hospital for 20 additional days for rehabilitation. During that time, she was treated for aspiration pneumonia and *C. difficile* infection. Later, she was transferred to a local hospital for rehabilitation due to general weakness.

## Discussion and conclusions

To the best of our knowledge, this report is the first to describe a case of SFTS-associated encephalopathy with detection of an identical SFTSV from the patient’s plasma and CSF and from a tick collected from the patient. Data on the transmission of SFTS from ticks to humans are uncertain [7, 8]. Among patients with SFTS, uncertainty about whether a tick bite has occurred is more common than a clear history of a tick bite [7]. However, SFTSV is prevalent (2.1–5.4%) in *H. longicornis* ticks collected from domestic animals in the areas where patients with SFTS live, which suggests that this tick is a major vector of SFTSV [1, 2]. In addition, SFTSV has been identified in ticks collected from the bodies of two patients with laboratory-confirmed SFTS [8]. However, phylogenetic analyses were not performed in those cases, making it impossible to know whether the ticks were the causative vectors of the disease or were incidental. In this report, the SFTSV detected from the tick was identical to that detected in the patient’s blood and CSF. Our findings demonstrate a strong link between an SFTSV-infected *H. longicornis* specimen and a patient with SFTS.

Although CSF examination did not reveal pleocytosis in this case, SFTSV was identified in a follow-up CSF sample by RT-PCR; this finding is consistent with a previous report of SFTSV detection in CSF, despite the absence of pleocytosis and normality of CSF protein and glucose findings [9]. In addition, on day 6 of admission, the viral load was higher in CSF than in plasma. The patient’s slow mental recovery during hospitalization may be related to the high viral load in CSF and prolonged adherence of the tick. Previous studies have suggested that other bunyaviruses are neurotropic [10], but this property has not been well studied for SFTSV, and the pathogenic mechanism of SFTSV as a cause of encephalitis remains to be identified.

There are some considerations that although samples were handled with great caution, possibility of cross contamination of the tick lysate by amplicons from the patient sample remains and a chance of the patient being the source of the SFTSV, transmitting the virus to the tick, since high level of viremia was detected in the patient. However, incubation period of SFTS is known as 1~ 2 weeks [11] and *H. longicornis* feeds for extended period of time on their hosts, varying from at least 4 days to 19 days [12] depending on life stage. Based on these facts the tick found on the patient’s body may have been the one responsible for transmitting the SFTSV to the patient.

In conclusion, an identical SFTSV retrieved from the blood and CSF of a patient with SFTS and the tick that bit her provide further evidence of SFTS viral transmission from *H. longicornis* to human, which strongly suggesting that the tick was the vector for SFTS.

## Abbreviations

ALT: Alanine transaminase
AST: Aspartate aminotransferase
CSF: Cerebrospinal fluid
GCS: Glasgow coma scale
LDH: Lactate dehydrogenase
M: Medium
PBS: Phosphate-buffered saline.
RT-PCR: Reverse-transcription polymerase chain reaction
SFTS: Severe fever with thrombocytopenia syndrome
SFTSV: Severe fever with thrombocytopenia syndrome virus
